# Gut microbiota analysis reveals microbial signature for multi-autoimmune diseases based on machine learning model

**DOI:** 10.3389/fmicb.2025.1660775

**Published:** 2025-09-25

**Authors:** Tianfeng An, Shuya Zhang, Jinjin Li, Hui Wang, Li Chen, Yiran Shi, Jingyi Wang, Sirui Han, Ruoxi Wang, Linyuan Wang, Zijing Huan, Ruiqi Yang, Desong Hao, Yanfang Liu, Xuehua Liu, Chao Yuan

**Affiliations:** ^1^The School of Public Health, Tianjin Medical University, Tianjin, China; ^2^The School of Medicine, Nankai University, Tianjin, China; ^3^Department of General Practice, Tianjin Union Medical Center, The First Affiliated Hospital of Nankai University, Tianjin, China; ^4^Tianjin Key Laboratory of Environment, Nutrition and Public Health, Tianjin Medical University, Tianjin, China; ^5^Center for International Collaborative Research on Environment, Nutrition and Public Health, School of Public Health, Tianjin Medical University, Tianjin, China; ^6^Department of Toxicology and Sanitary Chemistry, School of Public Health, Tianjin Medical University, Tianjin, China; ^7^The School of Clinical Medical, Tianjin Medical University, Tianjin, China

**Keywords:** gut microbiota, autoimmune diseases, machine learning, microbial signature, auxiliary diagnostic evaluation

## Abstract

**Introduction:**

Human microbiota is a major factor contributing to the immune system, offering an opportunity to develop non-invasive methods for disease diagnosis. In some research on Autoimmune Diseases (AIDs), gut microbiota variation has been observed. However, there remains a paucity of research that explores the potential of gut microbiota as a microbial signature for the classification and diagnosis of multi-AIDs.

**Methods:**

In this study, we analyzed 1,954 gut microbiota sequencing datasets from public databases collected from 1,043 patients with 10 AIDs to identify common or unique microbial signatures for AIDs through differential abundance testing and machine learning techniques. We evaluated five popular algorithms: Random Forest (RF), Support Vector Machine (SVM), K-Nearest Neighbors (KNN), Multilayer Perceptron (MLP), and eXtreme Gradient Boosting (XGBoost) models. Five-fold cross-validation and grid search were used to select the model parameters.

**Results:**

After comparing the performance of five models, the XGBoost model showed superior performance and achieved an area under the receiver operating characteristic curve (AUROC) ranging from 0.75 to 0.99 when predicting different diseases in the test set. At a specificity of 0.7 to 0.96, the sensitivity ranged from 0.66 to 1. By correlating the top 77 microbiota genera with the disease phenotypes, 126 significant associations were identified [false discovery rate (FDR) < 0.05]. We improved the detection accuracy and disease specificity for AIDs and revealed microbiota features specific to 10 different AIDs. Moreover, we found changing trends in shared microbiota features across some AID phenotypes, such as Crohn's Disease (CD) and Ulcerative Colitis (UC). At the same time, opposite changing trends were observed in the shared microbial signatures, such as Psoriasis and Myasthenia Gravis (MG). These results suggest that specific gut microbiota genera may affect the host immunity and induce different AID phenotypes.

**Discussion:**

This research holds potential for clinical application in the auxiliary diagnostic evaluation and monitoring of treatment responses. Simultaneously, it provides important clues for research on the characteristics of the intestinal immune microenvironment for different AIDs.

## Introduction

The gut microbiota represents a highly intricate community of microorganisms, encompassing bacteria, archaea, and eukaryotes, that inhabit the intestine. It is estimated that in approximately 100 trillion cells (Ley et al., 2006), the symbiotic microbes within the human gut exceed the number of host cells by at least an order of magnitude and possess a substantially larger repertoire of unique genes when compared to the host genome. Overall, the intestinal microbiota is composed of approximately 500–1,000 species, which belong to only a limited number of known bacterial phyla (Zoetendal et al., 2008). Emerging research suggests that the gut microbiota demonstrates vast enzymatic potential and plays a pivotal role in modulating diverse aspects of host physiology, such as pathogen resistance, host immunity, and metabolic processes (Sommer and Bäckhed, 2013; Belkaid and Hand, 2014; Blaser and Falkow, 2009).

The gut microbiota plays a crucial role in maintaining a delicate equilibrium between host defense and immune tolerance (Yang et al., 2021). The microenvironment within the gut is shaped by complex and intricate interactions between the gut microbiota and the local innate immune system (Piccioni et al., 2022; Gensollen et al., 2016). Optimally, the interaction between the immune system and microbiota functions in harmony, integrating both the innate and adaptive components of immunity to select, calibrate, and terminate immune responses, thus preserving homeostasis. Nevertheless, this immune balance between the gut flora and host is not invariably stable. A variety of pathologies affecting humans, including allergies, autoimmune disorders, and inflammatory conditions, stem from the inability to regulate misdirected immune responses against self-antigens, microbiota-derived antigens, or environmental antigens (Zoetendal et al., 2008; Jiao et al., 2020; Mu et al., 2017; Wang et al., 2015).

Autoimmune diseases (AIDs) occur when an individual's immune system mistakenly attacks its own tissues, with an estimated global incidence of approximately 3–5% (Miller et al., 2012; Ramos-Casals et al., 2015). Human microbiota is thought to be a crucial factor in the development of autoimmunity, as alterations in the microbial composition can result in the breakdown of immune tolerance (Belkaid and Hand, 2014; Shamriz et al., 2016). Systematic analysis of the human gut microbiota holds promise for developing non-invasive diagnostic methods for major AIDs. With the emergence of next-generation sequencing (NGS), novel strategies have been developed to investigate the association between gut microbiota dysregulation and AIDs. These strategies entail bioinformatic analysis to characterize the microbial compositions of samples, including the identification of microbial taxa and their relative abundance. Moreover, in case–control studies, researchers have attempted to identify differentially abundant microbial taxa as potential disease biomarkers (Quince et al., 2017; Liu et al., 2021; Knight et al., 2018). This approach has been applied to a variety of AIDs, such as systemic lupus erythematosus (SLE), rheumatoid arthritis (RA), inflammatory bowel diseases (IBD), and systemic sclerosis (SS) (Hevia et al., 2014; Chen et al., 2022; Volkmann et al., 2016; Andréasson et al., 2016; da Silva Brito et al., 2022; Vich Vila et al., 2018; Vaahtovuo et al., 2008; Chen et al., 2016).

However, the existence of shared microbial signatures across diverse diseases and overlapping gut microbiota signatures among most health states poses challenges for accurate diagnosis when using single-disease models, which can result in misclassification (Gacesa et al., 2022). To address this, multi-class diagnostic models have been developed to predict disease-specific signatures across the microbiota, enabling more accurate diagnostic purposes (Khan and Kelly, 2020; Su et al., 2022; Li M. et al., 2023). Machine learning (ML) classifiers are often employed for disease diagnosis, either using gut microbiota data alone or in conjunction with clinically relevant features, to differentiate patients from healthy controls (Ghannam and Techtmann, 2021; Marcos-Zambrano et al., 2021; Curry et al., 2021). ML-based gut microbiota classifiers have been developed for a variety of diseases, including inflammatory bowel disease (IBD), liver cirrhosis (LC), autism spectrum disorder (ASD), Alzheimer's disease (AD), and numerous others (Jiang et al., 2021; Qin et al., 2014; Oh et al., 2020; Kartal et al., 2022; Nagata et al., 2022; Dan et al., 2020; Liu et al., 2019; Li et al., 2019).

In the present study, we conducted a comprehensive meta-analysis of multiple AIDs. A total of 1,954 samples were used in this study. These diseases spanned 10 major disease categories. To comprehensively characterize the gut microbiota in relation to 10 AIDs, namely rheumatoid arthritis (RA), ankylosing spondylitis (SpA), multiple sclerosis (MS), psoriasis, Crohn's disease (CD), ulcerative colitis (UC), celiac disease (CeD), myasthenia gravis (MG), systemic lupus erythematosus (SLE), and type 1 diabetes (T1D), we utilized gut microbiota sequencing data to evaluate the abundance of taxonomic units. Furthermore, we developed a machine learning multi-classification model for the diagnosis of multi-AIDs and identification of microbial signatures that are either common across or specific to these 10 AIDs.

## Materials and methods

**Figure 1:**
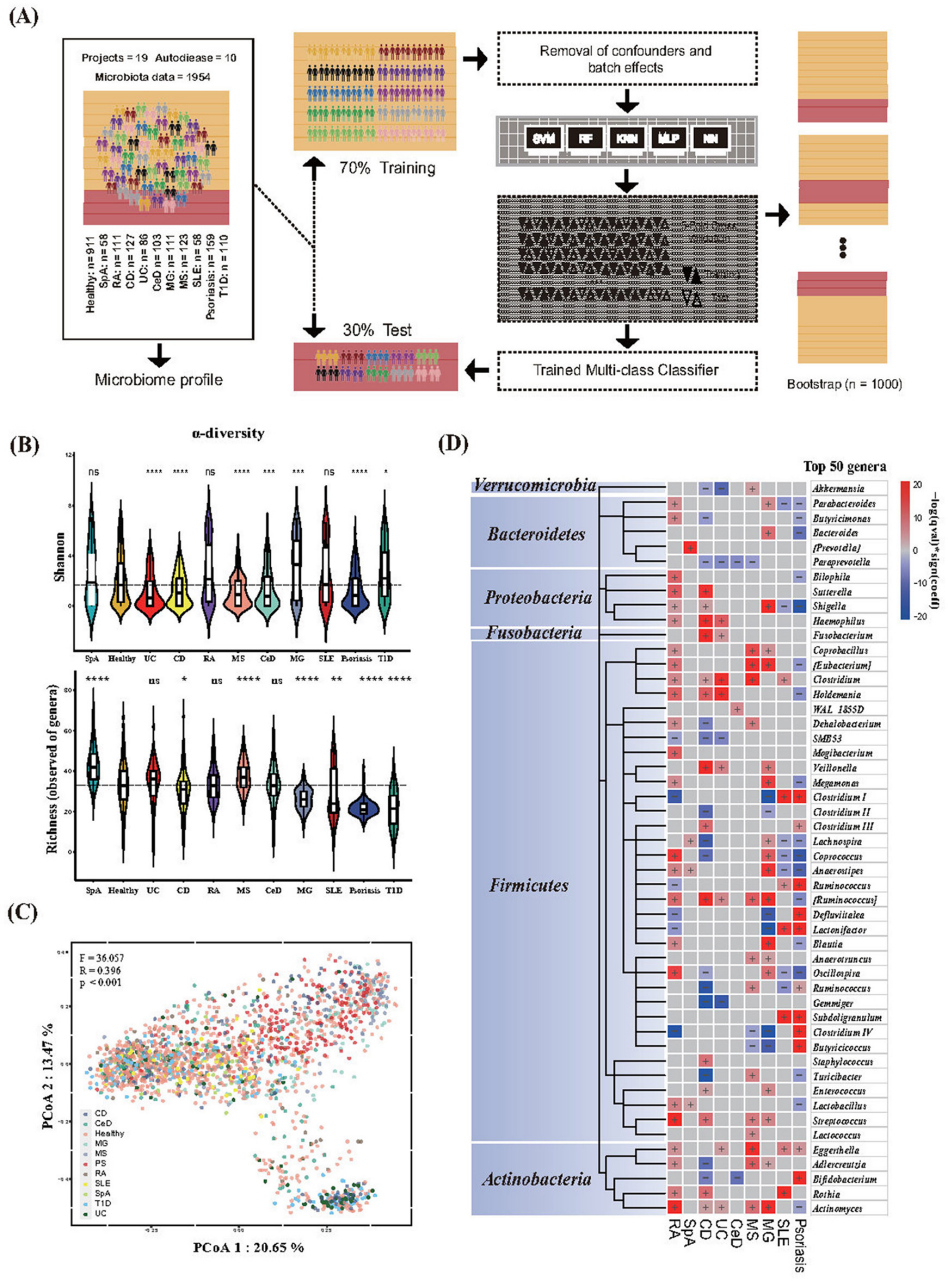
Gut microbiota features of healthy individuals and those with diseases. **(A)** Framework for dataset partitioning, model training, and validation. **(B)** Alpha diversity metrics for Shannon diversity and richness (number of microbial genera) in different phenotypes. The centerline represents the median, and the boundaries of the box represent the upper and lower quartiles. Kruskal–Wallis test. **(C)** Bray–Curtis dissimilarity-based principal coordinate analysis (PCoA) of genus-level. Each data point represented an individual sample. The *F*-, *R*-, and *P*-values were calculated using PERMANOVA with 999 permutations. **(D)** Heat map of microbial genera associated with different phenotypes. The top 50 microbial genera with the highest numbers of associations were identified. The significance (*p*-value) of the associations was calculated using MaAsLin 2, and FDR was determined using the Benjamini–Hochberg correction. Associations are colored by direction of effect (red, positive; blue, negative), with associations significant at FDR < 0.05, marked with a plus (positive correlations) or negative (negative correlations), respectively. RA, Rheumatoid Arthritis; SpA, Spondylitis ankylosing; CD, Crohn's disease; UC, Ulcerative colitis; CeD, Celiac disease; MS, Multiple sclerosis; MG, Myasthenia gravis; SLE, Systemic lupus erythematosus; T1D, Type 1 diabetes. For all the *P*-values in the figure, *p < 0.05; **p < 0.01; ***p < 0.001; ****p < 0.0001.

The main framework for dataset partitioning, model training, and validation of this research was shown in Figure 1A.

### Data collection

A comprehensive list of human autoimmune disease-related case–control studies on gut microbiota was performed in public databases, including NCBI BioProject (https://www.ncbi.nlm.nih.gov/bioproject) and GMrepo (a curated database of human gut metagenomes; https://gmrepo.humangut.info) (Dai et al., 2022; Wu et al., 2020). A total of 1,954 gut microbiota sequences on 10 different autoimmune diseases (Supplementary Table S1, run-level data including 1,043 cases and 911 controls) were collected. AIDs included RA, SpA, MS, Psoriasis, CD, UC, CeD, MG, SLE, and T1D. The specific inclusion and exclusion criteria are as follows: our inclusion criteria included: (1) Study on 16S rRNA amplicon sequencing of gut microbiota with complete disease phenotype metadata. (2) Case–control studies with at least nine valid samples in each case and control group. (3) No antibiotics or probiotic supplements were administered in the past 3 months. The exclusion criteria were as follows: (1) post-treatment follow-up after medication. We divided these 10 diseases into 7 categories, including 3 digestive system diseases, 2 AIDs of the nervous system diseases, 2 musculoskeletal diseases, 1 endocrine system disease, 1 connective tissue disease, and 1 skin disease, according to the NCBI Medical Subject Headings (MeSH, https://meshb.nlm.nih.gov/) database and Human Disease Ontology (DO) database (Schriml et al., 2022).

### Sequencing data extraction and microbiota profiling

We extracted all SRA_ID (listed in Supplementary Table S1) from the NCBI SRA database (https://www.ncbi.nlm.nih.gov/sra) (Katz et al., 2022) or European Nucleotide Archive (ENA) (Harrison et al., 2021) and obtained related information on samples from the NCBI BioSample database (https://www.ncbi.nlm.nih.gov/biosample). Raw sequencing data (FATSQ files) were then downloaded using the SRA toolkit, and metadata, including age, gender, and country, were collected. Trimmomatic (Bolger et al., 2014) was used to trim the reads and to remove sequencing vectors and low-quality bases. Reads shorter than 50 base pairs were discarded after trimming. To preprocess the sequences, we used QIIME (2023.5) (Bolyen et al., 2019) to demultiplex and quality-filter the data. Representative sequences and their abundances were extracted using the feature table (McDonald et al., 2012a) to generate tables containing amplicon sequence variants (ASVs). Taxonomic assignment of the individual dataset was classified against the Greengenes database (version 13.8123) (McDonald et al., 2012b). Genus-level relative abundance results were retained for subsequent analyses. Subsequently, samples with only two or fewer taxa were excluded from subsequent analyses. Additionally, to minimize the noise caused by low-abundance taxa, we filtered out those with a relative abundance of < 0.001 across all samples.

### Microbiota analysis

All statistical analyses were performed using R, version 4.2.2. The ggpubr package (https://github.com/kassambara/ggpubr) was used to perform non-parametric statistical testing between groups and to account for multiple hypothesis testing corrections when necessary. Significant differences in alpha diversity (Shannon index and richness) were determined using the non-parametric Kruskal–Wallis test. Differences in beta diversity (Bray–Curtis distance matrix calculated using the relative abundances of microbial genera) were determined by PERMANOVA using distance matrices (adonis) in the adonis function of the vegan R package with 999 permutations. Principal coordinate analysis (PCoA) based on beta-diversity was used to visualize the clustering of samples based on their genus-level compositional profiles. To adjust these findings for other factors that may affect microbiota, we used microbiota multivariable associations with linear models (MaAsLin) to identify compositional differences while adjusting for age, sex, and country. To account for the sequencing batch effects of all samples treated at different periods, we used the *adjust_batch* function implemented in the “MMUPHin” R package using project ID as the controlling factor before model development. Detailed information on the effectiveness of the batch effect removal in this study is shown in Supplementary Figure S1.

### Classification model for multiple-autoimmune-diseases

Binary sub-cohorts were composed of one AID phenotype and its corresponding healthy control, resulting in a total of 10 binary subgroups. The random forest (RF) model was chosen as the binary classifier because its classification performance has been shown to outperform other methods for microbiota data (Pasolli et al., 2016). The RF model was first trained on a randomly selected training set (5-fold stratified cross-validation) and then applied to the withheld test set to assess the final performance. This process was repeated 20 times to obtain a distribution of RF prediction evaluations for the test set, and the mean AUROC value was calculated.

Multi-class models can be implemented in Python 3.8 using standard libraries that are publicly available, including pandas (2.0.3), numpy (1.22.4), scikit-learn (1.3.1), and matplotlib (3.7.3). For each phenotype, the samples were randomly divided into training (70%, *n* = 1,368) and test (30%, *n* = 586) sets. Random forest (RF), support vector machine (SVM), K-nearest neighbors (KNN), multilayer perceptron (MLP), and eXtreme Gradient Boosting (XGBoost) were used as classifier models to diagnose different phenotypes based on the taxonomic profiles at the genus level of the gut microbiota. We employed a 5-fold cross-validation and grid search to select the optimal model parameters for the RF, SVM, KNN, and XGBoost models. For the MLP models, we implemented the default settings provided by Scikit-learn. Finally, we evaluated the performance of the five models on the test dataset as the final performance for predicting different diseases. The highly ranked and frequently selected microbiota features were considered predictive signatures for further interpretation.

### Model validation and performance evaluation

The area under the receiver operating characteristic curve (AUROC) is a widely used metric for evaluating the performance of classification models and provides a comprehensive assessment of the model's sensitivity and specificity trade-off at different thresholds. The range of AUROC values is typically explained from 0.5 to 1, with a higher value indicating a better ability to distinguish between different classes of samples. The area under the precision-recall curve (AUPRC), as a complementary assessment, considers the trade-offs between precision (or positive predictive value) and recall (or sensitivity) and is more robust for imbalanced datasets. The AUPRC ranges from 0 to 1, with a value of 0 signifying no positive examples identified and a value of 1 indicating perfect identification of all positive examples. In addition, for a more comprehensive evaluation of the model's performance, we employed the F1 score. It is a metric that considers the precision and recall of a model. The F1 score ranges from 0 to 1, with a higher value indicating better overall performance of the model.

We employed the bootstrap method to address the data imbalance and obtain more robust performance evaluations for each model. The bootstrap method involves iteratively resampling the training data, training a new model, and evaluating the model 1,000 times (Wang et al., 2023; Ning et al., 2025). The performance of the model was calculated as the average performance of the individual models developed using the bootstrap method. Bootstrap methods can considerably reduce overfitting in the developed models. To identify the most discriminative features among many bacterial genus features, minimize model complexity, and enhance computational efficiency and interpretability, we generated a learning curve relating the number of features to the model performance.

### Sensitivity analysis

Considering the potential influence of the three factors, “gender,” “age,” and “geographical location,” on the gut microbiota, we conducted the sensitivity analysis. For the factor “geographical location,” >75% of the samples in this study were collected from the United States (746) and China (724). We evaluated our model using country-based stratification. For factors “gender” and “age,” the Kruskal–Wallis test was conducted to verify the significance of the differences in the abundance of the corresponding genus in the gut microbiota among different age groups and different genders regarding the AID phenotype. Age groups: Juvenile (3–20), Young Adult (21–45), Older Adult (46–65), and Elderly (65+); Gender groups: Female and Male (Supplementary Table S1, “Sensitivity analysis”).

## Results

### Summary of available data

A total of 1,954 gut microbiota sequencing data (1,043 AIDs cases and 911 non-disease controls; sequences based on Illumina platforms) were collected from the NCBI database based on 19 case–control studies. These data could contain up to 127 cases and 247 controls; however, most studies were conducted on a limited number of samples, with median sizes of 55 cases and 48 controls, respectively. The fecal samples for the gut microbiota sequencing data were mainly collected from five continents and 13 countries, most of which were from the United States of America (38.18%), China (32.44%), and Canada (11.36%) (related information was shown in Supplementary Table S1, “Data availability”).

### Gut microbiota features across different phenotypes

Ecological indices may not be robust indicators for distinguishing disease from healthy status, which results in changes in the structure of the gut microbiota. First, we aimed to determine the differences in the composition of the gut microbiota among the different AIDs. Compared to healthy controls, significant differences in bacterial diversity (Shannon) and richness (number of genera) were observed in AIDs, except for RA. The Shannon index of the digestive system AIDs decreased. Moreover, we found that both indices (Shannon and richness) varied across phenotypes (Figure 1B). The results of microbial diversity among different phenotypes are consistent with a recent study on multi-class disease diagnosis based on gut microbiota and meta-analysis (Su et al., 2022; Gupta et al., 2020). Beta diversity based on Bray–Curtis dissimilarity showed significant differences in gut microbiota composition among individuals with different phenotypes (*R* = 0.396; *F* = 36.057; *p* < 0.001) (Figure 1C). We then used the linear model of MaALin2 after adjusting for age, sex, country, and technical confounders to explore the associations of microbial composition at the genus level with disease phenotypes. We found 192 significant associations between the 11 phenotypes and 62 bacterial taxa at the genus level (FDR < 0.05). Among the 62 genera, > 67.7% were significantly associated with two or more diseases. The genera *Haemophilus, Veillonella, Eggerthella*, and *Rothia* were positively associated with AIDs in our results, whereas the opposite was observed with the genera *Paraprevotella, SMB53*, and *Gemmiger* (Figure 1D, Supplementary Table S1 “Gut microbiota data”).

### Development of a gut microbiota-based diagnosis model

**Figure 2:**
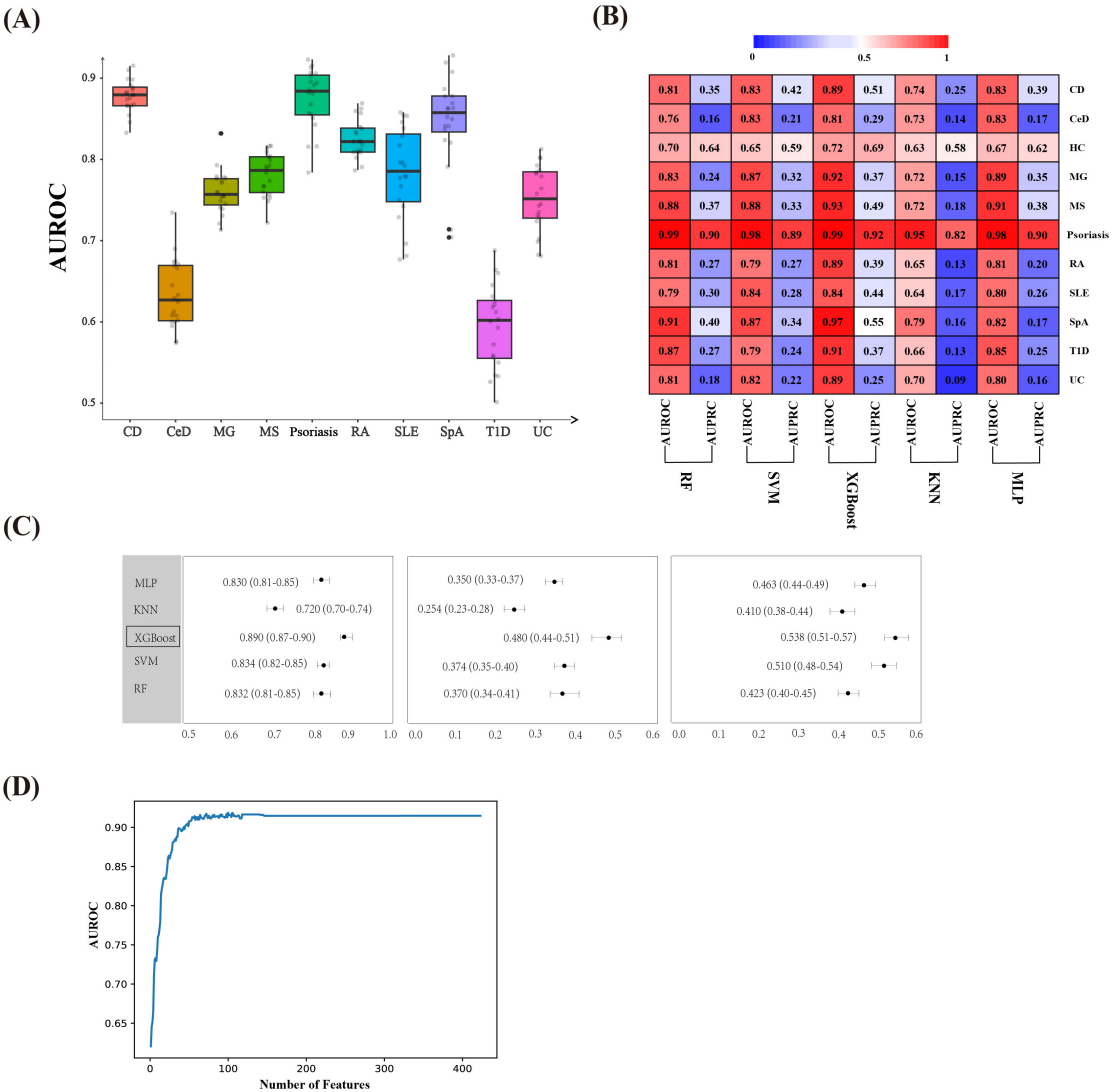
Comparison of different classifiers for phenotype classification using fecal microbiota data at the genus level. **(A)** Area under the receiver operating characteristic curve (AUROC) of random forest binary classifiers for disease vs. healthy control discrimination. **(B)** Performance across models was measured using the AUROC and area under the precision-recall curve (AUPRC) for predicting one phenotype vs. all others in the test set. **(C)** Mean AUROC, AUPRC, and F1-score, along with their corresponding 95% confidence intervals (95% CI), were calculated for the five machine learning models using the bootstrap method. **(D)** Learning curve of the relationship between the number of microbial genera and AUROC values. RA, Rheumatoid Arthritis; SpA, Spondylitis ankylosing; CD, Crohn's disease; UC, Ulcerative colitis; CeD, Celiac disease; MS, Multiple sclerosis; MG, Myasthenia gravis; SLE, Systemic lupus erythematosus; T1D, Type 1 diabetes; RF, Random forest; SVM, support vector machine; KNN, K-nearest neighbors; MLP, multilayer perception; XGBoost, eXtreme Gradient Boosting.

The binary classifier of the RF model based on the microbiota could significantly distinguish between healthy and most AIDs (Figure 2A), which indicated that AIDs had different degrees of intestinal flora disturbance compared with the control. To further investigate the discriminatory ability of the gut microbiota in various AIDs and to distinguish between AIDs and healthy controls, we constructed multi-class classifiers.

To select the best multi-class machine learning algorithm, we evaluated five popular algorithms: RF, SVM, KNN, MLP, and XGBoost models. Five models achieved a mean AUROC of all phenotypes of 0.72–0.89 (Figure 2B), suggesting that muti-class disease classification based on the gut microbiota was feasible. Amongst them, the XGBoost multi-class model had an optimal performance and achieved a mean AUROC of 0.89 [interquartile range, IQR (0.87–0.90)], a mean AUPRC of 0.48 [interquartile range, IQR (0.44–0.51)], and a mean F1-score of 0.538 [interquartile range, IQR (0.51–0.57)] for different disease phenotypes in the test set (Figure 2C). Therefore, the XGBoost multi-class classifier was used for further analysis.

**Figure 3:**
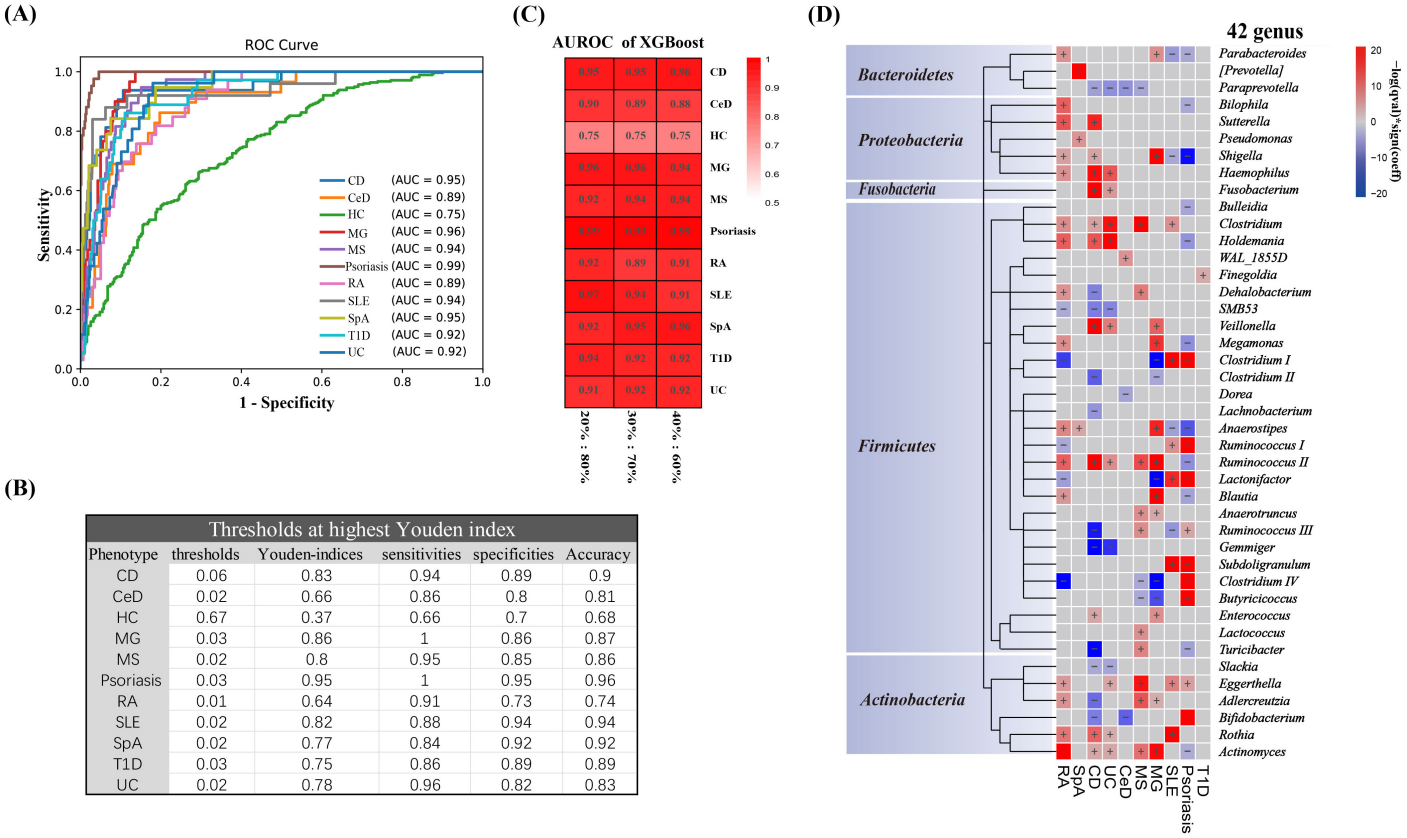
Gut microbiota-based XGBoost model for multi-class disease diagnosis. **(A)** Receiver operating characteristic curve of the XGBoost multi-class model, including 77 microbial genera. **(B)** Performance metric details of the trained XGBoost multi-class classifier for classifying one phenotype from all others using genus-level fecal microbiota data in the test set. **(C)** AUROC of the XGBoost multi-class model across different split ratios. **(D)** Microbial genera associated with different phenotypes. The top 77 microbial genera that contributed to the XGBoost multi-class classifier were clustered by taxonomy. The significance (*p*-value) of the associations was calculated using MaAsLin 2, and FDR was determined using the Benjamini–Hochberg correction. Associations are colored by direction of effect (red, positive; blue, negative; *p* < 0.05), with associations significant at FDR < 0.05, marked with a plus (positive correlations) or minus (negative correlations), respectively. RA, Rheumatoid Arthritis; SpA, Spondylitis ankylosing; CD, Crohn's disease; UC, Ulcerative colitis; CeD, Celiac disease; MS, Multiple sclerosis; MG, Myasthenia gravis; SLE, Systemic lupus erythematosus; T1D, Type 1 diabetes.

The XGBoost model is constructed using a complete training set. The importance rankings of all features at the genus level were obtained by leveraging this model. Subsequently, the features were incorporated in descending order of importance. For each step of adding a feature, the AUROC value of the model was computed, thereby generating a learning curve that depicted the relationship between the number of features and model performance (Figure 2D). The results indicate that the model performance reached a plateau when 77 features were employed. At this stage, a further increase in the number of features did not lead to a significant improvement in performance. Consequently, the first 77 features were selected as the input variables for the final model. The AUROC values for most phenotypes exceed 0.9 (Figure 3A). The macro-AUROC value was 0.9 [IQR (0.88–0.91)], indicating superior performance compared to the binary classifier we trained. This classifier proved to be valuable for effectively distinguishing AIDs based on features derived from the gut microbiota. At the optimal Youden's index threshold, the sensitivities of our XGBoost multi-class model range from 0.66 to 1 at specificities of 0.70 to 0.95 for different diseases with accuracy from 0.74 to 0.94, highlighting good diagnostic performance (Figure 3B). For example, our model achieved an AUROC of 0.95, CD with a sensitivity of 0.94, and a specificity of 0.89 (Figures 3A, B). To better characterize the XGBoost model, we compared its performance on datasets with different split ratios and obtained similar results, which indicated that the model exhibited high stability and good predictive capability without the risk of overfitting (Figure 3C).

### Associations between microbiota features and phenotypes

Next, we correlated the top 77 bacterial genera that contributed to the model with the different disease phenotypes. Among the 77 genera, 126 significant associations were found between 42 genera and the different disease phenotypes. These 42 genera belonged to the phyla *Firmicutes* (28 genera), *Actinobacteria* (6 genera), *Proteobacteria* (5 genera), *Fusobacteria* (1 genus), and *Bacteroidetes* (3 genera) (Figure 3D). Among the selected bacterial genera, significant associations were found between the phenotypes and several genera, except for T1D (genera only). From the perspective of AID phenotypes, CD (23 genera), RA (21 genera), and psoriasis (20 genera) were the three phenotypes with the highest number of related genera (FDR < 0.05). However, in SpA and CeD, fewer related genera were found (n < 5). From the perspective of the gut microbiota, the genera *Actinobacteria* and *Ruminococcaceae II* correlated with the largest number of AID phenotypes (six AID phenotypes). The genera *Shigella, Clostridium*, and *Eggerthella* also correlated with many AID phenotypes (five AID phenotypes). These genera could serve as shared microbiota features, which may suggest an association with AID phenotypes except for T1D. The genus *Dorea, Lachnobacterium, WAL_1855D, Bulleidia, Pseudomonas*, and the special genus *Prevotella* are only associated with one AID phenotype. This may imply that specific changes in the gut microbiota are related to the corresponding AID phenotype. The genera *Clostridium, Eggerthella, Haemophilus, Fusobacterium, Subdoligranulum*, and *Rothia* positively correlated with several AID phenotypes. However, *Paraprevotella, SMB53, Clostridium II, Gemmiger*, and *Slackia* were negatively correlated with several AID phenotypes. Only *Fusobacterium*, which belongs to *Fusobacteria*, was positively correlated with two inflammatory bowel diseases (CD and UC), indicating a potential microbiota feature of inflammatory bowel diseases.

Interestingly, we noted a higher degree of similarity in microbial alterations between diseases within the same system, such as inflammatory bowel diseases (CD and UC). Among these two phenotypes, 11 genera (approximately 50%) were shared between these two phenotypes, exhibiting similar trends in microbial changes. CeD, also an autoimmune disease of the digestive system, presents a completely distinct profile of related genera. Analogous results have also been reported in Psoriasis and SLE. Although shared microbiota features were relatively scarce, eight genera with similar trends in microbial changes were identified. In autoimmune diseases of the nervous system (MS and MG) and musculoskeletal system (RA and SpA), such similarities in microbial changes were not observed. On the other hand, in some AID phenotypes, alterations in the microbiota showed an opposite trend compared to the healthy group. For example, in Psoriasis and MG, ten microbiota features were shared between the two AID phenotypes, including the genera *Actinobacteria, Butyricicoccus, Clostridium IV, Blautia, Lactonifactor, Shigella, Anaerostipes, Clostridium I, Parabacteroides*, and *Ruminococcus II*. However, opposite trends were observed. Similar findings were obtained in a comparison of psoriasis and RA. These results suggest that, in different AIDs, the gut microbiota microenvironment may possess completely opposing characteristics.

### Sensitivity analysis

**Figure 4:**
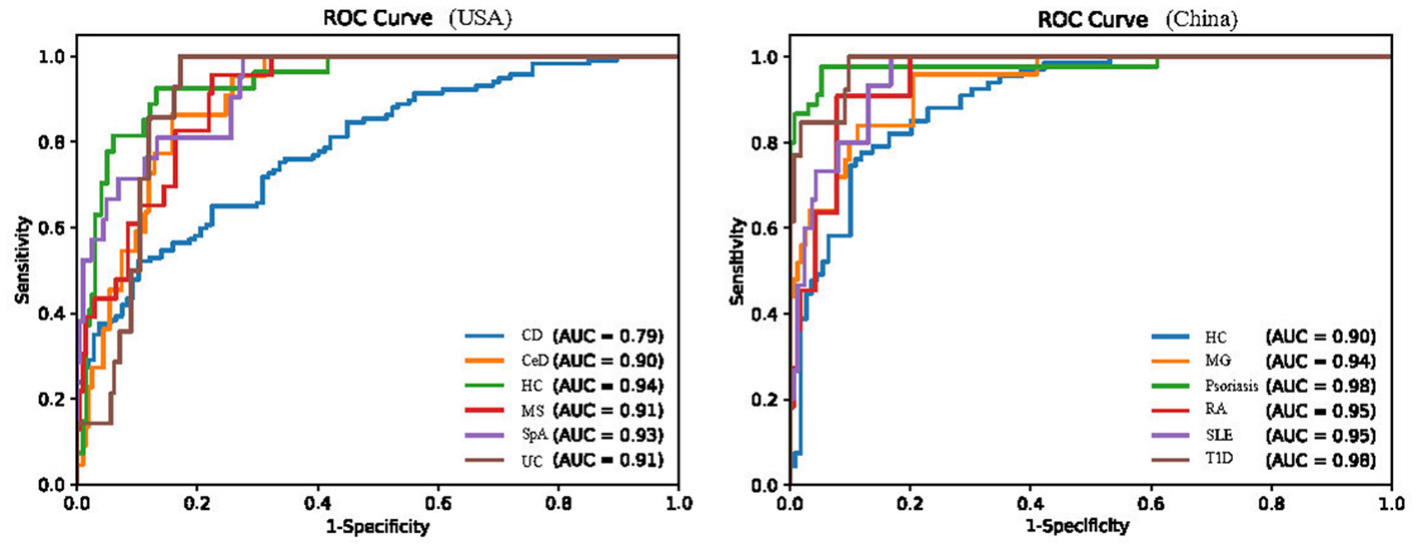
Performance of the XGBoost multi-class classifier for AIDs samples stratified by country. RA, Rheumatoid Arthritis; SpA, Spondylitis ankylosing; CD, Crohn's disease; UC, Ulcerative colitis; CeD, Celiac disease; MS, Multiple sclerosis; MG, Myasthenia gravis; SLE, Systemic lupus erythematosus.

The data collected for this study were sourced from multiple countries. More than 70% of the study participants were from the United States and China. The model's performance was evaluated using country-based stratification, revealing consistent results (Figure 4). Moreover, the model attained a mean Area Under the Receiver Operating Characteristic Curve (AUROC) of 0.90 (Interquartile Range, IQR: 0.88–0.93) in the United States and 0.91 (0.88–0.93) in China, respectively. Given the potential influence of “gender” and “age” on the gut microbiota, we performed a stratified analysis based on age and gender (Supplementary Table S1, “Sensitivity analyses”). The sensitivity analysis results indicated that “Geographic location,” “Age,” and “Sex” are not the primary factors affecting classification outcomes.

## Discussion

The human gut microbiota has assumed growing significance as a biomarker for non-invasive disease screening and as a target for disease intervention owing to its profound association with human diseases. In this study, we comprehensively aggregated publicly available datasets of gut microbiota sequencing. Moreover, we integrated microbiota features among diverse AIDs and utilized advanced and reproducible machine learning approaches that are highly relevant to clinical practice. Overall, this study demonstrated the feasibility of using a multi-classifier based on gut microbiota for the identification of AIDs. We propose that this multi-class model shows great potential for clinical applications in auxiliary diagnosis evaluation and assessment of the efficacy of interventions. Simultaneously, it offers crucial clues for the investigation of the characteristics of the intestinal immune microenvironment in different AIDs that primarily affect various body sites.

Our analysis of the gut microbiota revealed microbiota features associated with the 10 AIDs. Most of these findings are consistent with previous research on the correlation between the gut microbiota and AIDs. For example, *Actinomyces* spp., which act as both pathogens and constituents of human microbiota, have been reported to be enriched in patients with IBD or colorectal cancer (Yachida et al., 2019; Pittayanon et al., 2020). *Bifidobacterium* spp. are important probiotics, particularly during infancy. In the infant gut microbiota, a relationship between *Bifidobacterium* spp. and allergies has been identified, which plays a vital role in immune modulation and tolerance (Cukrowska et al., 2020; Gavzy et al., 2023; Derrien et al., 2022). *Adlercreutzia* spp. and *Clostridium* spp. were decreased in the gut microbiota of patients with CD (Leibovitzh et al., 2022). *Haemophilus* spp., a type of gut microbiota, has been reported to be associated with several immune-related disorders, including RA, IBD, and Hashimoto's thyroiditis (Liu et al., 2022; Zhang et al., 2015; Dunalska et al., 2023). Similarly, in our study, we also detected significant enrichment of this bacterial genus in patients with RA and IBD. Meanwhile, in studies exploring the gut microbiota of patients with type 2 diabetes (T2D), Parkinson's disease, and Alzheimer's disease, differences in the genus *Haemophilus* were observed between the disease groups and healthy control groups (Li Z. et al., 2023; Letchumanan et al., 2022). *Eggerthella* spp. and *Prevotella* spp., as typical gut microbiota signatures, were reported to be enriched in SLE, which is consistent with our results (Bixio et al., 2024; Yao et al., 2023). Genus *Eggerthella* levels have been implicated in inflammatory diseases, especially human gut *Eggerthella lenta* in AIDs and other conditions (Plichta et al., 2021; Xiang et al., 2021; Chang and Choi, 2023). *Veillonella* spp. are closely related to the genus *Clostridiales*, which are recognized as probiotic organisms in the host (Furusawa et al., 2013). Other studies have indicated that *Veillonella* spp. act as inflammophilic pathobionts, thriving in an inflammatory milieu, and possess the inherent ability to stimulate IL-6-mediated inflammation (van den Bogert et al., 2014). Notably, in a study of the gut microbiota in autoimmune hepatitis (AIH), the genus *Veillonella* was identified as the key genus strongly associated with the disease (Wei et al., 2020; Yuming et al., 2024). The genus *Fusobacterium* was significantly increased in IBD in our study, and previous research has found a similar association (Volkmann et al., 2016; de Paiva et al., 2016; Hong et al., 2023; Cornejo-Pareja et al., 2020; Islam et al., 2022). In particular, *Fusobacterium nucleatum* exhibits a wide range of characteristics under certain conditions. It can adhere to a large number of phylogenetically unrelated bacterial species, potentially leading to the translocation of non-invasive, yet pro-inflammatory species across the compromised intestinal epithelium, thereby exacerbating the disease state (Uitto et al., 2005; Strauss et al., 2011). Given that the AIDs included in this study preferentially targeted distinct body organs, it is unsurprising that we detected differences, as prior studies have reported analogous conclusions (Forbes et al., 2016). In our study, the number of microbiota features corresponding to each AID phenotype was lower than the number of significant differential microbiota abundances found in existing studies based on basic case–control studies. This is attributable to the fact that our model was constructed using data from 10 AIDs. In addition to considering the differences between AIDs and controls, it also accounts for potential disparities among the various AID phenotypes. Consequently, the selected microbiota features are relatively fewer. However, some of our findings deviated from those of previous studies. For example, previous studies have indicated that *Prevotella* spp. are more abundant in the early/preclinical stages of RA, whereas *Bifidobacterium* spp. are less abundant. Nevertheless, our study did not yield similar results (Ajith and Anita, 2025). Our findings also identified several bacterial genera that were scarcely observed in previous investigations of the 10 AIDs. *Rothia* spp. have been mentioned in research on treatable periodontitis, endocarditis, and joint infections (Michels et al., 2007; Verrall et al., 2010; Colombo et al., 2012). Our study revealed a potential association between the genus *Rothia* and SLE, RA, and IBD, which has not been previously reported. *Paraprevotella* spp., encompassing numerous opportunistic pathogens, has only been reported in the fecal samples of patients with rare AIDs, such as Behcet's disease (BD), Vogt-Koyanagi-Harada (VKH) disease, and the dextran sulfate sodium-induced IBD model (Ye et al., 2020, 2018; Sabater et al., 2022). In our study, there was a significant reduction in *Paraprevotella* in MS, CD, UC, and CeD. In the colorectal cancer mouse model, the *Paraprevotella* spp.-derived metabolite agmatine triggered inflammation to promote colorectal tumorigenesis through the Wnt signaling pathway (Lu et al., 2024). This might be the key mechanism by which *Paraprevotella* spp. regulates the gut immune microenvironment in various AIDs. Although disease phenotypes were not observed in this study, the genus *Slackia* was found to be more abundant in patients with APECED-associated hepatitis (APAH) (Chascsa et al., 2021). The discovery of these bacterial genera suggests that multi-classifiers based on deep machine learning are more conducive to uncovering gut microbiota features that have not been easily discerned in previous studies across a broader range of data.

As described in the “Results” section, we observed a higher degree of similarity in microbial alterations among the two inflammatory bowel diseases (CD and UC). Among these two phenotypes, 11 shared genera exhibited similar trends in terms of microbial changes. Analogous findings were also noted in Psoriasis and SLE, where eight shared genera with similar trends in microbial changes were identified. In contrast, in the nervous system, AIDs (MS and MG) and musculoskeletal system (RA and SpA) AIDs, such similarities in microbial changes were not detected. IBD is a consequence of the interaction between the host and microorganisms, encompassing intestinal microbial factors, abnormal immune responses, and a damaged intestinal mucosal barrier. CD and UC are two subtypes of IBD that may have similar intestinal immune microenvironments, leading to numerous shared microbiota features with comparable trends of microbial changes (Danne et al., 2024; Anderson et al., 2021). Psoriasis and SLE are chronic autoimmune diseases that affect multiple organs. Although their specific pathogenic mechanisms differ, they all involve abnormal activation of immune cells, abnormal secretion of cytokines, and T cell-mediated inflammation (Griffiths et al., 2021; Durcan et al., 2019). In our results, eight genera, *Eggerthella, Subdoligranulum, Ruminococcus, Lactonifactor, Anaerotruncus, Clostridium, Parabacteroides*, and *Shigella*, shared microbiota features with similar trends. *Eggerthella* spp. can induce intestinal Th17 activation by lifting inhibition of the Th17 transcription factor Rorγt through cell- and antigen-independent mechanisms (Bixio et al., 2024). *Subdoligranulum* spp. are arthritogenic strains that trigger RA and can stimulate Th17 cell expansion in mice (Zhu et al., 2020). In colorectal cancer, *Ruminococcus* spp. can maintain the immune surveillance function of CD8+ T cells through its metabolic characteristics (Schirmer et al., 2019). The remaining three genera were also associated with other AIDs, including MS, RA, and IBD (Bixio et al., 2024; Ajith and Anita, 2025; Anderson et al., 2021; Yousefi et al., 2023; Caruso et al., 2020).

On the other hand, in some AID phenotypes, alterations in the microbiota exhibited an opposing trend compared to the healthy control group. For example, in Psoriasis and MG, 10 microbiota features were shared between these two AID phenotypes, including the genera *Actinobacteria, Butyricicoccus, Clostridium IV, Shigella, Blautia, Lactonifactor, Anaerostipes, Clostridium I, Parabacteroides*, and *Ruminococcus II*; however, opposite trends of change were found. Similar results were also found in Psoriasis and RA (opposite-trend-genera: *Actinobacteria, Clostridium IV, Blautia, Clostridium I, Ruminococcus I, Ruminococcus II, Parabacteroides, Bilophila Lactonifactor, Megamonas, Anaerostipe, Holdemania*, and *Shigella*). Th17 cells play a crucial role in the pathogenesis of AIDs. Although specific pathological regions vary among different AID phenotypes, Psoriasis, RA, and MG involve an imbalance in Th17/Treg cells (Alexander et al., 2022; Zhang et al., 2024; Szekanecz et al., 2021). Almost all the above-mentioned genera with opposite trends have been reported to be associated with Th17 cells or T-cell-related inflammatory responses, except for the genera *Holdemania* and *Bilophila* (Bixio et al., 2024; Ajith and Anita, 2025; Sun et al., 2023; Schirmer et al., 2019; Lian et al., 2024; Yu et al., 2025; Lu et al., 2024; Zou et al., 2024). Numerous investigations have demonstrated that the gut microbiota can exert an impact on host immunity via its metabolites. This process, in turn, affects AID phenotypes, and this mechanism represents a key strategy for treating AIDs (Yang and Cong, 2021). Our findings indicate that, within distinct AIDs, the gut microbiota microenvironment may exhibit completely opposing characteristics. The variations in AID phenotypes may be attributed to the influence of specific gut microbiota patterns on the host immune response process. Combined with the role of genetic factors, this leads to an imbalance of Th17/Treg cells in different regions of the body, ultimately giving rise to the emergence of corresponding pathological phenotypes.

### Strengths and limitations

The strengths of this study include the use of gut microbiota data covering a variety of disease phenotypes (10 AIDs), including gut microbiota sequencing data from almost 2,000 participants. However, this study has some limitations that should be acknowledged. First, we aimed to include gut microbiota sequencing data covering the widest possible range of AIDs and the corresponding healthy controls. However, microbiota sequencing data in public databases often lack relevant information, such as host comorbidities, diet, BMI, and treatment/medication conditions. In our study, only sex (70.5%), age (86.9%), and geographical location (100%) were comprehensively available. Previous research has shown that age and geographical location influence gut microbiota composition, and in some diseases, such as systemic lupus erythematosus (SLE), sex also plays a role (Pang et al., 2023; Bradley and Haran, 2024; He et al., 2018). Therefore, we conducted sensitivity analyses based on these three factors (Figure 4 and Supplementary Table S1, “Sensitivity analyses”). Most genera showed significant differences; however, in the ≥65 age group, some genera did not, likely due to the limited sample size rather than age-related effects. Moreover, because of the limitations of publicly available sequencing data (16S limitations), our analyses were restricted to the genus level, and species-level analyses could not be performed. Second, all datasets were retrospectively obtained, and the classifier was not validated in an external prospective cohort. The complex phenotypes of AIDs, prolonged sample collection periods, and limited availability of prospective data in public databases preclude such validation. Future studies should involve collaboration with relevant clinical teams to prospectively collect fecal samples, utilize gut metagenomic sequencing to obtain deep-level microbiota information, assess the classifier's predictive value for disease progression or prognosis, and enhance its clinical applicability. Finally, the data were cross-sectional, limiting our ability to determine the temporal sequence and causality between abnormal gut microbiota and the onset of AIDs.

## Conclusion

In conclusion, this study offers a comprehensive analysis of the composition of stool microbiota in AIDs. Our findings show that the composition of gut microbiota changes in rheumatoid arthritis (RA), spondyloarthritis (SpA), Crohn's disease (CD), ulcerative colitis (UC), celiac disease (CeD), multiple sclerosis (MS), myasthenia gravis (MG), systemic lupus erythematosus (SLE), and psoriasis. These changes are notably associated with varying degrees of gut dysbiosis. Moreover, through differential abundance testing and machine learning techniques, we identified several microbial signatures that exhibit consistently higher or lower abundances in AIDs patients than in healthy controls. Subsequent research is imperative to delve into the specific roles and functions of this genus within the host. This is crucial for establishing causal associations in disease pathogenesis and for exploring their potential as targets for future therapeutic interventions.

## Data Availability

The data that supports the findings of this study are available in NCBI SRA database (https://www.ncbi.nlm.nih.gov/sra); accession numbers listed in Supplementary Table S1.

## Supplementary material

Supplementary Figure S1 **(A)** Detailed information on the effectiveness of batch effect removal. Shrinkage of the batch mean parameters. *X*-axis: estimated batch mean parameter (Gamma); *Y*-axis: batch mean parameter after shrinkage (Gamma-shrunk). Shrinkage can stably adjust the batch mean parameters. **(B)** Original/adjusted mean abundance. *X*-axis: overall mean; *Y*-axis: mean values from the different batches. The correction process makes the originally scattered batch expressions more compact and closer to the overall average expression, thereby significantly reducing the batch effect.

Supplementary Table S1 Comprehensive information on autoimmune diseases (AIDs) was collected for this study. Details of gut microbiota sequencing data and related samples (Bioproject_ID, SRA_ID, AIDs, Age, Gender, Country) were listed in sheet 1, “Data availability.” Ten autoimmune diseases (AIDs) are listed in Table 1. Microbial abundance results of gut microbiota sequencing data and related ID were shown in sheet 2, “Gut microbiota sequencing.” Sensitivity analysis on two factors (gender and age) was shown in sheet 3, “Sensitivity analysis.” For each genus, the Kruskal–Wallis test was conducted to verify the significance of the differences in the abundance of the corresponding genus in the gut microbiota among different age groups and sexes regarding the AID phenotype.
