# Supplementary figures and images for: Gut microbiota analysis reveals microbial signature for multi-autoimmune diseases based on machine learning model
Source: Front Microbiol. 2025 Sep 25;16:1660775. doi: 10.3389/fmicb.2025.1660775 (PMC12507882; doi:10.3389/fmicb.2025.1660775)

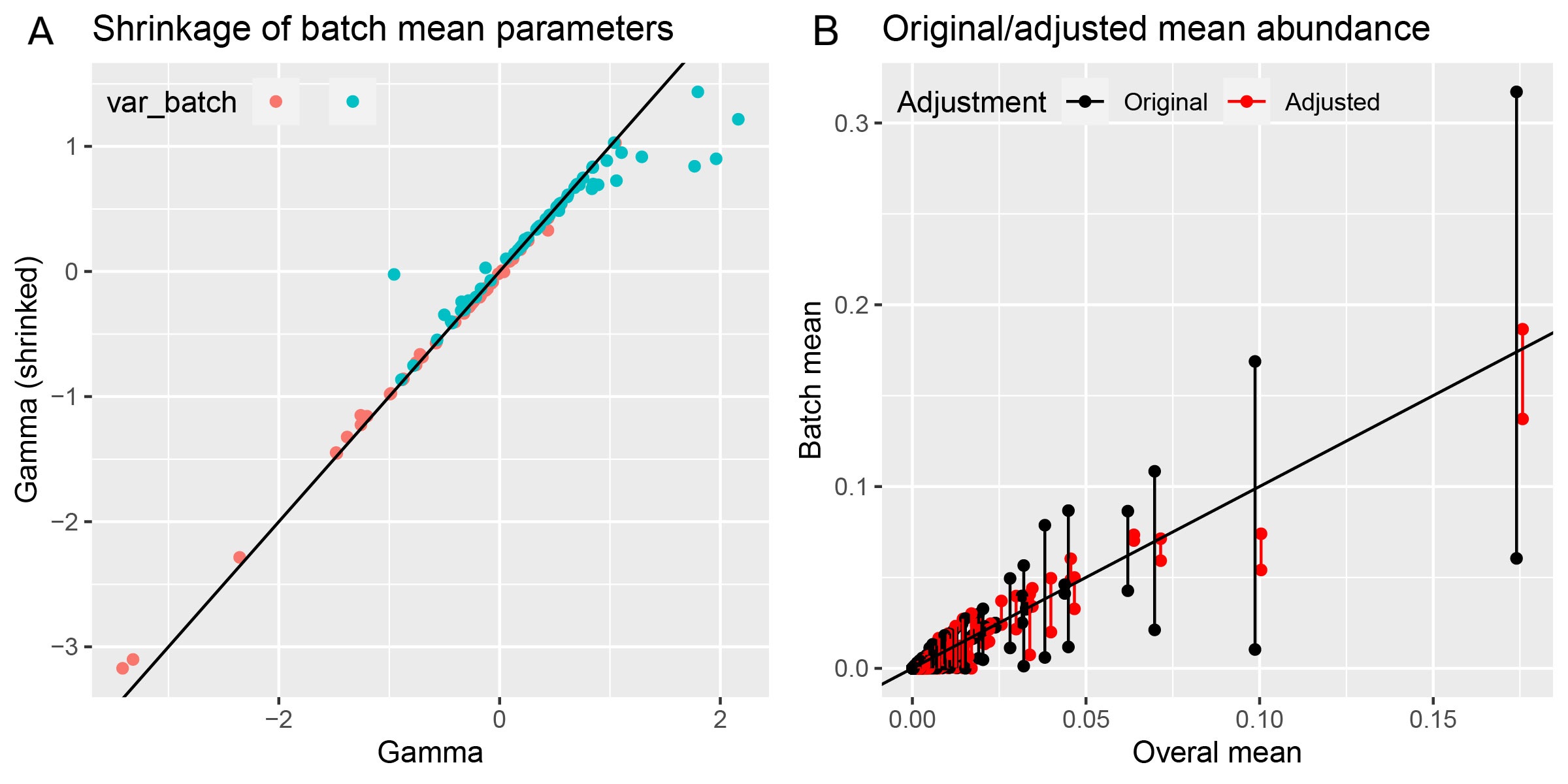

Supplement: Supplementary Figure S1 — (A) Detailed information on the effectiveness of batch effect removal. Shrinkage of the batch mean parameters. X-axis: estimated batch mean parameter (Gamma); Y-axis: batch mean parameter after shrinkage (Gamma-shrunk). Shrinkage can stably adjust the batch mean parameters. (B) Original/adjusted mean abundance. X-axis: overall mean; Y-axis: mean values from the different batches. The correction process makes the originally scattered batch expressions more compact and closer to the overall average expression, thereby significantly reducing the batch effect. [file Image_1.jpeg]
